# ACGA a Novel Biomimetic Hybrid Optimisation Algorithm Based on a HP Protein Visualizer: An Interpretable Web-Based Tool for 3D Protein Folding Based on the Hydrophobic-Polar Model

**DOI:** 10.3390/biomimetics10110763

**Published:** 2025-11-12

**Authors:** Ioan Sima, Daniela-Maria Cristea, Laszlo Barna Iantovics, Virginia Niculescu

**Affiliations:** 1 Computer Science Department, Babes-Bolyai University, 400114 Cluj-Napoca, Romania; ioan.sima@ubbcluj.ro (I.S.); virginia.niculescu@ubbcluj.ro (V.N.); 2 Informatics, Mathematics and Electronics Department, University ‘1 Decembrie 1918’ of Alba Iulia, 510009 Alba-Iulia, Romania; 3 Doctoral School of Letters, Humanities and Applied Sciences, George Emil Palade University of Medicine, Pharmacy, Sciences and Technology of Targu Mures, 540088 Târgu Mureș, Romania; 4 Department of Electrical Engineering and Information Technology, Faculty of Engineering and Information Technology, George Emil Palade University of Medicine, Pharmacy, Science, and Technology of Targu Mures, 540142 Târgu Mureș, Romania

**Keywords:** biomimetic genetic algorithm use with interpretability, benchmark problem for genetic algorithms, protein structure prediction, lattice model, combinatorial optimization, representation, stochastic optimization, visualisation

## Abstract

In this study, we used the hydrophobic-polar (HP) two-dimensional square and three-dimensional cubic lattice models for the problem of protein structure prediction (PSP). This kind of lattice reduces computational time and calculations, the conformational space from $9^{n}$ to $3^{n-2}$ for the 2D square lattice and $5^{n-2}$ for the 3D cubic lattice. Even within this context, it remains challenging for genetic algorithms or other metaheuristics to identify the optimal solutions. The contributions of the paper consist of: (1) implementation of a high-performing novel genetic algorithm (GA); instead of considering only the self-avoiding walk (SAW) conformations approached in other work, we decided to allow any conformation to appear in the population at all stages of the proposed all conformations biomimetic genetic algorithm (ACGA). This increases the probability of achieving good conformations (self avoiding walk ones), with the lowest energy. In addition to classical crossover and mutation operators, (2) we introduced specific translation operators for these two operations. We have proposed and implemented an HP Protein Visualizer tool which offers interpretability, a hybrid approach in that the visualizer gives some insight to the algorithm, that analyse and optimise protein structures HP model. The program resulted based on performed research, provides a molecular modeling tool for studying protein folding using technologies such as Node.js, Express and p5js for 3D rendering, and includes optimization algorithms to simulate protein folding.

## 1. Introduction

The molecular structure of a protein can be broken down hierarchically into four levels. The protein’s primary structure is its sequence of amino acids (AAs), the secondary structure is its locally folding pattern, the tertiary structure is the globally folded form, frequently as a globule, and its quaternary structure is its multimeric organization [1].

Protein folding (PF) is the physical process by which a protein chain is translated into its native three-dimensional (3D) structure, typically a “folded” conformation in which the protein becomes biologically functional. This process is fundamental because only in a good tertiary structure (native conformation) can the protein become biologically functional [2].

The algorithms must predict the correct native conformation and the purpose of PSP is to predict the tertiary structure from the primary structure. Due to its extreme complexity, several simplified protein models have been proposed, reducing the algorithms search space. Three approaches can be classified: (1) ab initio-starting only from the information contained in the primary structure of the proteins, (2) homology-using information from the primary structure and knowledge of the native conformations of similar sequences, and (3) protein threading (or fold recognition), using knowledge of the fold families [3,4].

The fundamental motivations of this work are: (1) Complexity of protein modelling-understanding protein structures is crucial to revealing biological functions and diseases associated with protein misfolding, such as Alzheimer’s disease [5]. The HP model provides a simplified yet robust framework for examining the basic principles of protein folding without the need for extensive computational resources. (2) Lack of accessible tools-most existing tools, such as Rosetta [6] or GROMACS [7], requires advanced programming knowledge and is difficult to use.

The main objective of this work was to provide researchers, students, and bioinformatics professionals to explore protein folding visually in three-dimensional space. Through its interface, HP Protein Visualizer understanding of the relationship between amino acid sequences and resulting 3D structures, an essential aspect of protein study and its applications in biotechnology and medicine.

**Contribution to PSP.** This paper describes a new biomimetic genetic algorithm used for solving PSP on the HP model. The algorithm considers, at all stages, populations formed of all configurations, without excluding the invalid (unfeasible) ones. A conformation is valid (feasible) if and only if it respects the self-avoiding walk (SAW) condition, which means that no two beads (i.e., amino acids represented as nodes placed on a 2D or 3D lattice) are placed in the same position on the lattice (thus avoiding overlaps).

Allowing invalid configurations in the populations is beneficial due to the chaotic behavior of the associated energy, increasing the chances of finding good partial solutions (valid configurations) generated through small changes applied to some invalid configurations.

Furthermore, the original approach includes, in addition to classical crossover (rotational) and mutation (rotational and diagonal) operators, the application of translation operators for both crossover and mutation operations.

An important and novel aspect of this work lies in the hybrid integration between the ACGA and the HP Protein Visualizer. The visual component offers interpretability of how genetic operators influence protein geometry and provides an interface for debugging, hypothesis testing, and exploratory purposes.

The remainder of the paper is organized as follows: Section 2 presents the related work in the domain of PSP, followed by Section 3, which describes the HP model and the PSP within heuristic techniques. Section 4 presents the hybrid algorithms, while Section 5 presents and analyzes the results obtained by applying the proposed algorithms to a set of popular benchmarks. Finally, Section 6 and Section 7 offer a conclusive analysis of the results and future work.

## 2. Related Work

According to the thermodynamic hypothesis [8], the native conformation is determined solely by the protein’s AAs sequence, and in this conformation, the protein reaches its minimum free energy. Even if a given protein has only one native conformation (the correct conformation), it can still fold into a multitude of other conformations, which are erroneous.

Simulated annealing (SA) and genetic algorithm (GA) were applied by Unger [9], Custodio [10], Jiang [11] and Cox [12,13]; the evolutionary Monte Carlo (EMC) method applies Liang and Wong [14].

Zhou et al. [15] present a combination of particle swarm optimization (PSO) and tabu search (TS) to the PSP, using the 3D HP lattice model, and Huang applied GA-based on Optimal Secondary Structures (GAOSS) on the 2D HP model [16].

In a previous study [17], Benitez et al. applied a hybridization of the parallel artificial bee colony (ABC) and GA to the 3D HP model with side chains to solve PSP on the Beowulf cluster.

Rashid et al. [18], evaluated different algorithms, including the Local Search (SS-Tabu), GA, and hybrid algorithms (Hybrid GA), on the five benchmark sequences. However, their algorithms performed poorly on large proteins (sequences with lengths greater than 100 AAs). In another study [3], the authors used the single-point crossover genetic operator and mutation with rotation and diagonal-pull-tilt moves. Additionally, in a different study [19], the authors created a multithreaded local search framework (SS-Parallel) that allowed all threads to execute the spiral search (SS-Tabu) and GA (selections and random-walk).

Over time, several models have been proposed to predict protein structures and explain the folding process. A very simple but efficient model is the HP model, which was proposed by Dill [20] and further developed in the following years [21]. PSP on this model belongs to the **ab initio** methods, where the proteins are represented as sequences of hydrophobic or non-polar (H), and hydrophilic or polar (P) characters arranged on a 2D or 3D lattice. The goal is to find a conformation that minimises the energy, typically defined by the number of non-consecutive H-H contacts.

In the study of [22], a mathematical model for the HP triangular lattice was applied to a GA, obtaining good results on small sequences. More recently, the authors [23] applied a GA on the 2D HP model on larger sequences (50 to 100 AAs), but the optimum energy was not attained [23].

The HP model uses a discretized space (lattice), where the amino acids (AAs) of the protein sequence are placed on the lattice nodes. The conformations are determined by the positions of the AAs, and they are differentiated by introducing a free-energy function, which is a score determined by these positions and not by the thermodynamic free energy [24,25]. However, even with this discretization, an exhaustive search is not feasible as PSP on this model has been proven to be an NP-complete problem [26].

To solve PSP on the HP model, heuristic techniques from different classes, such as Machine Learning (ML) [27], evolutionary algorithms (EA) [28,29], GA [2], and Monte-Carlo (MC) simulations have been applied. PSP on the HP model requires combinatorial chromosome encoding, and the energy function expresses chaotic behavior in the Devaney sense [30]. That is why the GA applied to PSP has issues with convergence and reaching the optimum.

Recent advances in deep learning have revolutionised protein structure prediction. AlphaFold [31], developed by DeepMind, has achieved unprecedented accuracy in predicting protein 3D structures from amino acid sequences. It uses a transformer-based neural network architecture trained on protein structures and sequences. AlphaFold [32] demonstrated near-experimental accuracy in the CASP14 challenge, making it a groundbreaking development in structural bioinformatics.

RoseTTAFold [33], developed by the Baker lab, is another deep learning-based method for protein structure prediction [34]. It uses a three-track network that considers sequences, distance maps, and coordinates simultaneously. RoseTTAFold is open-source and has been used for applications beyond structure prediction, including docking and design.

The ab initio is important since it gives meaning to PF, but it has not yet been completely solved. Therefore, our focus is on this ab initio variant. From a computational standpoint, the ab initio variant of the HP model addresses the PSP problem as a combinatorial optimization task in a discrete search space S, where each conformation s∈S corresponds to a self-avoiding walk (SAW) on a 2D or 3D lattice. The objective function E:S→R computes the energy of a conformation based on hydrophobic (H-H) contacts, and the goal is to find the conformation with minimum energy:

(1) $$s^{*}=argmin_{s\in S}E\left(s\right).$$

Unlike AlphaFold or RosettaFold, which rely on probabilistic models trained on large biological datasets and perform inference in high-dimensional continuous spaces, the ab initio HP model operates without external information, learning or evolutionary alignments. It explores a rugged, high-dimensional, discrete landscape where exact methods are infeasible due to NP-completeness.

Therefore, heuristic and metaheuristic approaches, such as genetic algorithms [35,36], are essential for navigating the vast configuration space.

There were some state-of-the-art GAs developed in the scientific literature for the HP lattice model, primarily focusing on sequence optimization and folding energy minimization. In this context, the development of a biomimetic hybrid Genetic algorithm is used with visualization tools to ensure interpretability, enabling dynamic evaluation of folding configurations, parameter control, augmented by local move operators and guided by visual heuristics.

Recent advances in protein modelling span a diverse landscape of tools and data regimes, ranging from cryo-EM map–guided reconstruction (e.g., ModelAngelo) to deep learning–driven template and fragment assembly (e.g., D-I-TASSER, I-TASSER-MTD), generative probabilistic approaches (e.g., DiffModeler), and hybrid experimental–computational assembly (e.g., DEMO-EM). In this context, the proposed ACGA algorithm occupies a complementary niche: it is a lightweight, interpretable, lattice-based approach particularly suited for benchmarking metaheuristics and for didactic exploration of protein folding principles. A direct empirical comparison with the aforementioned high-resolution tools is not attempted here, as they operate on different types of input data (e.g., experimental density maps, evolutionary alignments) and target atomistic structural resolution rather than coarse-grained lattice configurations. Readers are referred to the cited works for detailed descriptions of these complementary methods, and we note that a future extended study will explore hybrid strategies that integrate lattice-based heuristics such as ACGA with learned priors or experimental constraints. Short conceptual descriptions:

- ModelAngelo: a tool designed to build atomic models from cryo-electron microscopy (cryo-EM) density maps; its primary inputs are experimental density maps and sometimes partial sequence information, and it focuses on map-guided model building and refinement [37].
- D-I-TASSER: a DL enhanced successor to I-TASSER that integrates template-based modeling, multiple-sequence alignments, and predicted inter-residue distances/contacts to assemble 3D structures from sequence. It therefore combines evolutionary information with learned structural priors [38].
- DiffModeler: representative of the emerging class of diffusion-model-based structure generators that sample structures by iteratively denoising a probabilistic latent representation; such methods typically learn complex structural distributions and can be conditioned on sequence or other constraints [39].
- I-TASSER-MTD: an extension of the I-TASSER pipeline that emphasizes multi-template and template-threading strategies combined with fragment assembly; it highlights how classical template-based assembly remains competitive when templates exist [38].
- DEMO-EM (or DEMO-EMol) [40]: methods that use cryo-EM/experimental density together with docking and model-assembly procedures to reconstruct multi-domain or complex structures [41].

In order to place ACGA in this landscape, we suggest a brief conceptual comparison along the following axes:

1. Primary input data: sequence-only (HP/ACGA), experimental maps (ModelAngelo, DEMO-EM), MSAs/templates (D-I-TASSER, I-TASSER-MTD) or learned priors/latent samplers (DiffModeler).
2. Modelling paradigm: discrete combinatorial search on lattices (ACGA/HP), template and fragment-based assembly, gradient-based deep learning, or probabilistic generative sampling (diffusion).
3. Output and resolution: coarse-grained/topological models (HP lattice) versus all-atom coordinates suitable for functional interpretation (ModelAngelo, D-I-TASSER).
4. Resource and interpretability trade-offs: HP-lattice methods and ACGA are lightweight and interpretable (good for algorithmic benchmarking and pedagogy). In contrast, modern DL and experimental-map-driven tools require substantial data and compute but deliver high-resolution predictions.

## 3. Protein Structure Prediction on the Hydrophobic-Polar Model

PSP on the HP model involves three stages: (1) choosing a suitable type of lattice (e.g., 2D-square, 2D-trigonal, 2D-rectangular, 3D-cubic, 3D-Face-Centered Cubic); suitability is defined either by the appropriateness to the structure of real proteins or by the higher probability to reach a solution; (2) choosing an arbitrary energy function to model the protein conformation on the lattice, making it similar to the real protein’s native conformation (i.e., forming the hydrophobic kernel/kernels and the polar surface); and (3) creating a new algorithm or modifying an existing one that can solve the protein folding problem in this lattice-based setting.

Two AAs placed on adjacent nodes in the lattice can be either sequence neighbors or topological neighbors. Sequence neighbors are neighbors in the protein’s primary structure, while topological neighbors are positioned on nearby nodes to form contacts.

The arbitrary energy function should be defined to maximize the number of contacts between hydrophobic AAs. This can be achieved by assigning a value of free energy for each type of contact, as follows: e(H,H)=−1; e(H,P)=0; e(H,P)=0; e(P,P)=0. Specifically, H-H contacts are encouraged, while other types of contacts are neither encouraged nor penalized. An H-H contact $\left(h_{i},h_{j}\right)$ between the *i*-th and *j*-th AA from the original sequence can be formed only if |i−j|>2. Based on these criteria, the free-energy, *E*, of a conformation *c* is calculated as the sum of H-H contacts, taken with the minus sign (see Equation (2)):

(2) $$\begin{array}{c} E\left(c\right)=-\sum_{i,j=1,|i-j|>2}^{n}e\left(a_{i},a_{j}\right) \end{array}$$

where *n* is the sequence length. The negation is applied to ensure similarity with thermodynamic free energy.

For the basic HP model, PSP problems on square lattices have been proved to be NP-complete problems by Crescenzi [42] and Berger [26]. Based on these results, it has been concluded that all other problems on more general lattices and on three-dimensional ones are also NP-complete.

**Problem definition.** For computational reasons, a more formal and general definition of the k-dimensional problem is needed:

Let be Λ a geometrical lattice in $R^{k}$ and $HP=(h_{1},h_{2},\ldots ,h_{n})$ be a string of *H* and *P* letters ($h_{i}\in \left\{H,P\right\},i\in \bar{1,n}$). The lattice is defined by:

(3) $$\Lambda =\left\{\left.\sum_{i=1}^{k}b_{i}v_{i}\right|b_{i}\in Z\right\}$$

where $\left\{v_{1},v_{2}\ldots v_{k}\right\}$ form a base of a vector space $R^{k}$.

For the 2D square lattice (k=2) and 3D cubic lattice (k=3), the vectors $v_{1}$…$v_{k}$ have to satisfy the following two conditions: (1) $v_{i}⊥v_{j},i.j\in \bar{1,k}$, and (2) $v_{1}=\ldots =v_{k}=1$.

The *HP* string represents an AAs sequence in the HP model, where *H* corresponds to a hydrophobic AA, and *P* corresponds to a polar AA. To simplify the explanation, we refer to the modeled AAs as “beads.” In the sequence, each bead has exactly two neighbors, except for the first and last beads, which have only one neighbor (successor and predecessor, respectively). The distance between any two neighboring beads is equal to 1.

**Conformations.** A conformation is defined by the placement of the HP string on the lattice. The placement must adhere to the following restrictions: (1) only one bead can be placed in each position on the lattice; (2) the HP string cannot be broken; neighboring beads in the string should be placed in neighboring positions in the lattice.

A conformation corresponds to a walk in the lattice, representing the placement of the HP string on it. A conformation is feasible if and only if it respects self-avoiding walk condition, which means that no two beads are placed in the same position on the lattice.

There are two common types of encodings used to represent a conformation. These encodings specify directions to follow the walk. In the first type, known as absolute encoding, the fold directions are relative to the lattice. In the second type, known as relative encoding, the fold directions are relative to the conformation itself.

For the 2D square lattice, the two encodings are denoted as follows:

1. RULD string—use for absolute encoding, where R,U,L,D letters stand for right, left, up, and down, respectively;
2. SRL string—use for relative encoding, where *S* stands for straight, *R* for right, and *L* for left.

For the 3D cubic lattice, the two encodings are denoted as follows:

1. RULDFB string—use for absolute encoding, where R,U,L,D have the same meaning as in a 2D square lattice, and F,B letters stand for front and back directions, respectively;
2. SRLFB string—use for relative encoding, where the letters R,U,L,D retain their meaning from points above.

The free energy of a conformation is calculated based on the Equation (2). Free energy of a conformation is a function depending on the *HP* string, the encoding string and the lattice type, as can be seen in Equation (4).

(4) E(c)=f(HP_string,encoding_string,lattice_type)

**Problem requirement.** For a given sequence (*HP* string), find the optimal configuration for which the free-energy is minimal [43]. This implies that the *H* beads are positioned in the center of the lattice, and this configuration should directly correlate with the native configuration of the real protein. Therefore, the problem is a combinatorial optimization one [44,45], which can be solved using either deterministic or non-deterministic algorithms. In this case, we have chosen non-deterministic GA. For all the cases, the problem input is the HP string that represents the unfolded string of beads (Example: HPHPPHHPHPPHPHHPPHPH).

The output of the algorithm depends on the lattice type. For example, in the two-dimensional case, the output could be the RULD string (or SRL string), representing the folded string with the minimal energy configuration that satisfies the SAW condition. The objective function is defined by the energy equation (Equation (2)).

## 4. Optimisation Algorithms

Three different optimization techniques are used to find protein conformations with the lowest amount of energy.

### 4.1. Monte Carlo Simulation

**Figure 1:**
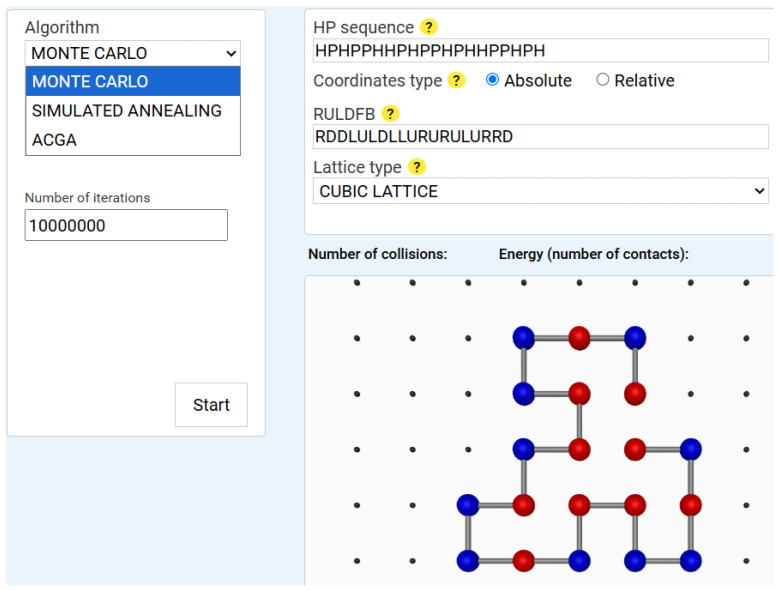
Monte Carlo simulation result for a 20-amino-acid *HP* sequence. *Note:* The figure shows the Monte Carlo algorithm after completing 10,000,000 iterations for a sequence of 20 amino acids. The input data for both the visualizer and the algorithm (*HP* sequence, Coordinates type, and Lattice type) are displayed in the upper-right panel. This panel also contains the optimal solution returned by the algorithm, represented by the *RULDFB* string. The canvas area displays the optimal conformation found by the algorithm. Hydrophobic amino acids, shown in red, are grouped in the central region of the protein, forming a hydrophobic kernel, while polar amino acids, shown in blue, are located in the peripheral region. The algorithm temperature is maintained constant at 1 degree.

Monte Carlo (MC) simulation is a stochastic method that explores the conformational space through random sampling [46]. It applies the Metropolis criterion [47] to decide whether to accept or reject a structural mutation based on energy change. Our implementation includes custom move sets, such as crankshaft motions, translational moves and pivot (rotational) moves. An example of a Monte Carlo execution result is displayed in Figure 1.

The system was evaluated with 10,000,000 iterations and a constant temperature of 1.0, which probabilistically accepts the new conformation, according to:

$$P\left(C\to C^{\prime }\right)=\left\{\begin{array}{cc} 1, & \mathrm{if}\;\Delta E\leq 0\\ e^{-\Delta E/T}, & \mathrm{if}\;\Delta E>0 \end{array}\right.$$

where $\Delta E=E_{\mathrm{new}}-E_{\mathrm{old}}$ (is the energy difference), and *T* is the constant temperature.

**Figure 2:**
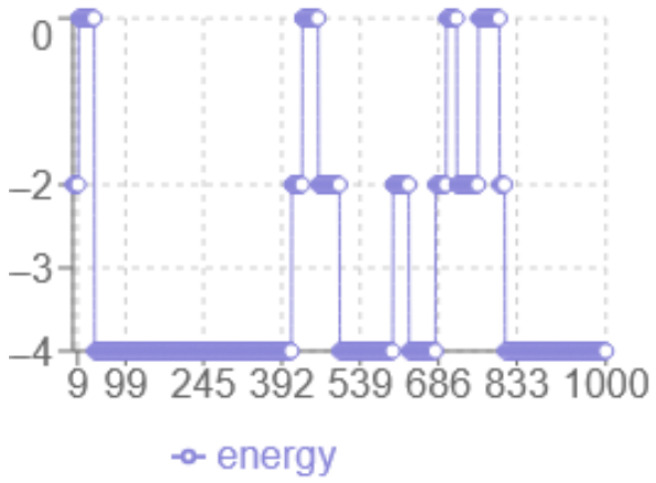
Monte Carlo Energy evolution.

The analysis of the energy profile in Figure 2 reveals the following characteristics: the *x*-axis corresponds to the iteration number, while the *y*-axis represents the energy level multiplied by two. The minimum energy achieved is −2 in this case. The algorithm displays a dynamic behaviour with significant fluctuations between 0 and −2, indicating more in-depth exploration of the solution space, with abrupt transitions that indicate the algorithm ability to escape local optima.

Algorithm 1 implements the Metropolis Monte Carlo (MC) strategy for exploring conformational space in the HP lattice protein folding model. The input consists of a binary *HP* sequence $S=(s_{1},s_{2},\ldots ,s_{n})$, where each monomer $s_{i}$ is either hydrophobic (*H*) or polar (*P*), along with the number of iterations and the lattice type (2D square or 3D cubic). The algorithm begins by initializing a valid conformation $C_{0}$ and computing its energy $E(C_{0})$, which serves as the initial reference.

At each iteration, a new candidate conformation $C^{\prime }$ is generated via a mutation operator. If $C^{\prime }$ is valid (i.e., free of overlaps), its energy $E^{\prime }$ is computed and compared to the previous energy $E_{i-1}$. If the energy difference $\Delta E=E^{\prime }-E_{i-1}$ is negative, the new conformation is accepted. Otherwise, it is accepted with a probability p=exp(−ΔE/T), where *T* is the system temperature. A random number r∈[0,1] determines acceptance based on *p*. If rejected, the previous conformation is retained.

The algorithm iteratively optimized the conformation, storing the best solution C* encountered. This approach allows escape from local minima. The output is the optimal conformation string encoded in *RULDFB* notation.
**Algorithm 1** Metropolis Monte Carlo Algorithm for HP lattice Model1:**Input**: S, iterations, lattice_type     ▹ HP sequence: $S=(s_{1},s_{2},\ldots ,s_{n})$ with $s_{i}\in \left\{H,P\right\}$2:**Output**: C*                                             ▹ RULDFB string (Best conformation found)3:Initialize a valid conformation $C_{0}$4:Compute energy of $C_{0}:E\left(C_{0}\right)$, $C*\leftarrow C_{0}$5:$E_{0}\leftarrow E\left(C_{0}\right)$6:**for** i=1 to iterations **do**7:    $C^{\prime }\leftarrow Mutation\left(C_{i-1}\right)$                                        ▹ Generate a new conformation, $C^{\prime }$8:    **if** $C^{\prime }$ is valid **then**                                                                   ▹ Check for overlaps9:        Compute energy $E^{\prime }\leftarrow E\left(C^{\prime }\right)$10:        $\Delta E\leftarrow E^{\prime }-E_{i-1}$11:        **if** ΔE≤0 **then**12:           Accept $C^{\prime }$: $C_{i}\leftarrow C^{\prime }$, $E_{i}\leftarrow E^{\prime }$, $C*\leftarrow C^{\prime }$13:        **else**14:           $p\leftarrow \exp\left(-\frac{\Delta E}{T}\right)$                                          ▹ Compute acceptance probability15:           Generate random number r∈[0,1]16:           **if** r<p **then**17:               Accept $C^{\prime }$: $C_{i}\leftarrow C^{\prime }$, $E_{i}\leftarrow E^{\prime }$18:           **else**19:               Reject $C^{\prime }$: $C_{i}\leftarrow C_{i-1}$, $E_{i}\leftarrow E_{i-1}$20:           **end if**21:        **end if**22:    **else**23:        Reject $C^{\prime }$: $C_{i}\leftarrow C_{i-1}$, $E_{i}\leftarrow E_{i-1}$24:    **end if**25:**end for**26:**return**C*;

### 4.2. Simulated Annealing

**Figure 3:**
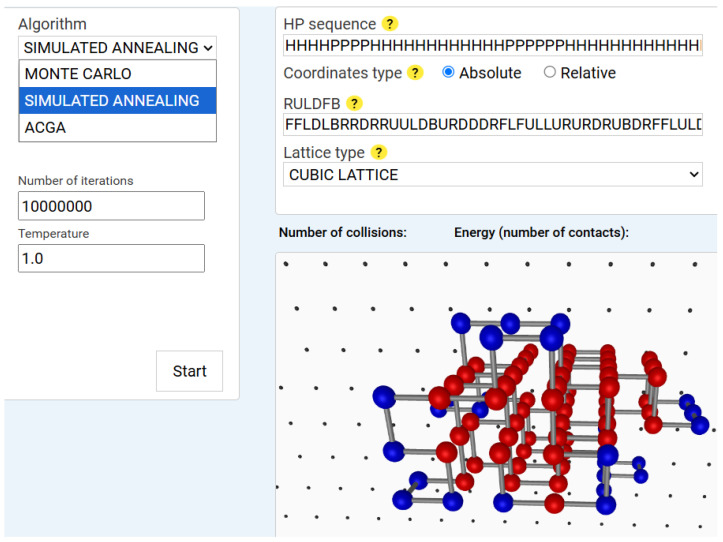
Simulated annealing simulation result for an 84-amino-acid *HP* sequence. *Note:* The user interface is almost identical to that of the Monte Carlo implementation, with the addition of the temperature parameter as an input for the algorithm. In this example, the initial temperature is set to 1 degree and gradually decreases throughout the iterations. The SA algorithm was executed on an *HP* sequence of 84 amino acids using a 3D cubic lattice. It is important to note that the lattice type parameter should not be changed from the graphical interface, since the difference between the 2D square and 3D cubic lattices lies only in the fact that, in the 3D case, the additional move directions *F* (Forward) and *B* (Backward) are in *RULDFB* conformation string.

Simulated Annealing (SA) extends the Monte Carlo approach by progressively lowering the temperature to reduce the acceptance probability of suboptimal solutions [48]. This mimics the annealing process in metallurgy. We use exponential cooling and adaptive temperature steps based on energy fluctuations. Figure 3 shows the results of a sample Simulated Annealing run.

The SA algorithm also ran for 1,000,000 iterations, starting from an initial temperature $T_{0}=1.0$, which was gradually decreased using an exponential cooling schedule:

$$T_{i}=T_{0}\cdot \alpha^{i},\;\;\alpha \approx 0.95$$

where $T_{i}$ is the temperature at iteration *i*; $T_{0}$ is the initial temperature; α is the cooling rate, and *i* is the iteration index.

**Figure 4:**
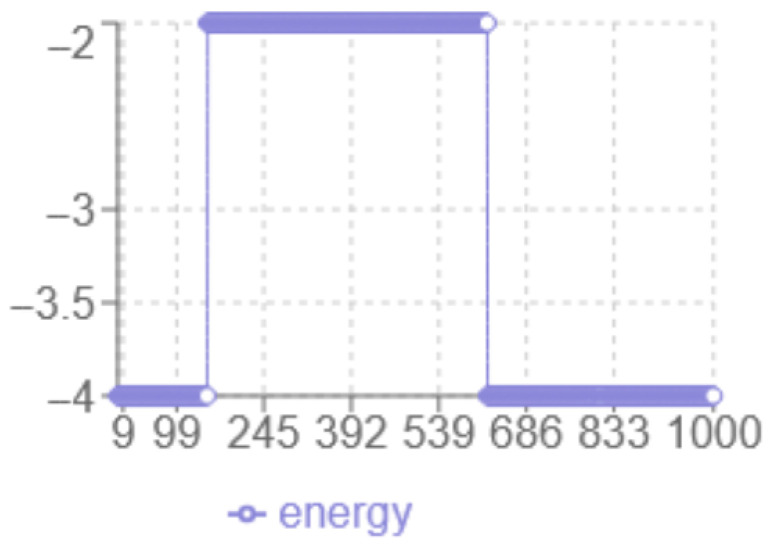
Simulated Annealing Energy evolution.

According to the energy analysis in Figure 4, the following observations were made: the *x*-axis corresponds to the iteration number, while the *y*-axis represents the energy level multiplied by two. The minimum energy achieved is −2. The dynamic behaviour shows a transition from −1 to −2 without abrupt jumps, and a noticeable plateau around −1, indicating prolonged exploration in that region. The final structure have increased linearity with fewer compact hydrophobic interactions, suggesting potential risks in a local optimum.

Algorithm 2 implements the SA optimisation strategy for exploring the conformational space of protein sequences modelled using the HP lattice framework. The input comprises an HP sequence $S=(s_{1},s_{2},\ldots ,s_{n})$, where each residue $s_{i}$ belongs to the set {H,P}, alongside the initial temperature $T_{0}$, cooling rate α, number of iterations, and the lattice type.

The procedure begins by generating a valid initial conformation $C_{0}$ and computing its associated energy $E(C_{0})$, which serves as the reference state. At each iteration, a new candidate conformation $C^{\prime }$ is produced via a random mutation applied to the current conformation. If $C^{\prime }$ is valid (i.e., free from overlaps and maintaining chain connectivity), its energy $E^{\prime }$ is evaluated and compared to the previous energy $E_{i-1}$.

If the energy difference $\Delta E=E^{\prime }-E_{i-1}$ is non-positive, the candidate is accepted unconditionally. Otherwise, it is accepted probabilistically with $p=\exp(-\Delta E/T_{i})$, where $T_{i}=T_{0}\cdot \alpha^{i}$ represents the temperature at iteration *i* according to an exponential cooling schedule. A random number r∈[0,1] is drawn, and $C^{\prime }$ is accepted if r<p; otherwise, the previous conformation is retained.

Throughout the iterative process, the algorithm tracks the best conformation $C^{*}$ encountered. This stochastic approach enables efficient exploration of the solution space, balancing exploitation of low-energy states with the ability to escape local minima. The final output is the conformation with the lowest energy identified during the search.

As illustrated in Algorithm 2, the key difference between SA and MMC lies in the temperature schedule. While both methods start with an initial temperature set to 1, SA applies an exponential decay across iterations (see line 13 in the algorithm). This adjustment balances the rate between exploration and exploitation. Initially, exploration is encouraged by accepting solutions or conformations with higher energy than the parent conformation. Towards the end of the algorithm, the acceptance probability decreases significantly, thereby promoting exploitation of the combinatorial search space.
**Algorithm 2** Simulated Annealing for HP Lattice Model1:**Input**: S, $T_{0}$, α, iterations, lattice_type                  ▹ S - HP String, $T_{0}$ - initial temperature2:Initialize a valid conformation $C_{0}$3:Compute energy of $C_{0}:E\left(C_{0}\right)$, $C*\leftarrow C_{0}$4:$E_{0}\leftarrow E\left(C_{0}\right)$5:**for** i=1 to iterations **do**6:    $C^{\prime }\leftarrow Mutation\left(C_{i-1}\right)$               ▹ Generate a new conformation, $C^{\prime }$ by random mutation7:    **if** $C^{\prime }$ is valid **then**                                      ▹ Check for overlaps and chain connectivity8:        Compute energy $E^{\prime }\leftarrow E\left(C^{\prime }\right)$9:        $\Delta E\leftarrow E^{\prime }-E_{i-1}$10:        **if** ΔE≤0 **then**11:           Accept $C^{\prime }$: $C_{i}\leftarrow C^{\prime }$, $E_{i}\leftarrow E^{\prime }$, $C*\leftarrow C^{\prime }$12:        **else**13:           $T_{i}\leftarrow T_{0}*\alpha^{i}$14:           $p\leftarrow \exp\left(-\frac{\Delta E}{T_{i}}\right)$                                               ▹ Compute acceptance probability15:           Generate random number r∈[0,1]16:           **if** r<p **then**17:               Accept $C^{\prime }$: $C_{i}\leftarrow C^{\prime }$, $E_{i}\leftarrow E^{\prime }$18:           **else**19:               Reject $C^{\prime }$: $C_{i}\leftarrow C_{i-1}$, $E_{i}\leftarrow E_{i-1}$20:           **end if**21:        **end if**22:    **else**23:        Reject $C^{\prime }$: $C_{i}\leftarrow C_{i-1}$, $E_{i}\leftarrow E_{i-1}$24:    **end if**25:**end for**26:**return**$C^{*}$

### 4.3. ACGA Algorithm

The proposed ACGA is a population-based method that evolves a set of protein conformations using selection, crossover, and mutation operations. The fitness function incorporates both energy minimisation and structural compactness metrics. ACGA involves the following steps:

1. Population Initialization: Firstly, a population of potential solutions is created. Each solution, referred to as a chromosome, is randomly generated, and it has an associated objective function called fitness.
2. Exploration Stage: The mutation and crossover operators are applied to a certain percentage of the chromosomes in the population, which are chosen randomly. These operators ensure the dispersion of the population in the space of possible solutions, promoting exploration of the solution space.
3. Through the selection operation, from a percentage of the individuals of the population, those with the best fitness are selected. In this way, a new population is created, usually statistically better, and this represents the next generation.
4. Exploitation Stage: Through the selection operation, a certain percentage of individuals with the best fitness are chosen from the population. This process creates a new population, which is usually statistically better and represents the next generation of potential solutions. The selection operation helps exploit the combinatorial space by favoring the fitter individuals for reproduction.

Steps (2) and (3) are iterated for a number of generations, allowing the population to evolve and improve over time. The mutation and crossover operators contribute to the exploration stage, while the selection operator contributes to the exploitation stage of the genetic algorithm.

**Chromosomes encoding.** We consider that the conformation of the modeled proteins can be encoded using either relative or absolute directions. We have chosen to use both in our approach to exploit the advantages of both encodings. The benefits of using relative encoding are as follows: (a) smaller combinatorial space compared to absolute encoding (relative—$3^{n}$; absolute—$4^{n}$); (b) implicit avoidance of returning the current AA to the previous AA position in the walk, during the creation of the initial population. (c) Mutation and crossover operators do not require modifications of letters that specify the next positions. On the other hand, absolute coding allows easy conversion into Cartesian coordinates.

Therefore, we have employed both the absolute and relative encodings, resulting in the following corresponding representations (strings):

- *HP* string
- *RULD* string—absolute 2D square
- *SRL* string—relative 2D square
- *RULDFB* string—absolute 3D cubic
- *SRLFB* string—relative 3D cubic

*HP* string is a sequence string. We will generically call *RULD* string, *SRL* string, *RULDFB* string and *SRLFB* string as conformation strings.

For computational efficiency reasons, the exploration is performed using the relative encoding, which reduces the conformation space from $4^{n-1}$ to $3^{n-1}$. However, the corresponding absolute encoding is also stored to enable easy and fast computation of the Cartesian coordinates. The size of the sequence string is equal to *n*, and the size of the conformation strings is equal to n−1, with each letter representing the relative successive direction in the conformation, where *n* is number of AAs of the sequence.

**Generation of the initial population.** The sequence string represents the input data, and the conformation string is the output of the ACGA algorithm. The primary structure of the protein is represented by the *HP* string sequence string) of *n* letters corresponding to the *n* AAs of a sequence.

Below are the steps for building a conformation (chromosome) using *SRL* string representation in the population initialization stage:

1. Set i = 1. Initialize *SRL*[i] = ‘S’.
2. i = i + 1. if i≤n−1 continue with the next step. Otherwise, the conformation is completely generated in the SRL string.
3. Choose a random direction ’d’ from the {*S*,*R*,*L* }.

For the 3D case, a similar construction is used based on the SRLFB string. Then, the SRL string is converted to the RULD string. The first letter, which is *S*, is always converted to the *R* letter. This fact reduces the 4-exponential combinatorial space by four times. After that, the RULD string is converted to an array of Cartesian coordinates. Based on these Cartesian coordinates, the number of collisions, the number of contacts, and the fitness are computed. The math formulas used for finding an H-H contact in the 2D square and 3D cubic lattices are as follows.

For the 2D square lattice:

If $abs\left(x_{i}-x_{j}\right)+abs\left(y_{i}-y_{j}\right)=1$, where $x_{i}$, $y_{i}$ are the Cartesian coordinates of the *i*-th AA, and $x_{j}$, $y_{j}$ are the Cartesian coordinates of the *j*-th AA, then there is a contact between the two AAs at positions *i* and *j*, $\forall i,j\in \bar{1,n}$.

For the 3D cubic:

If $abs\left(x_{i}-x_{j}\right)+abs\left(y_{i}-y_{j}\right)+abs\left(z_{i}-z_{j}\right)=1$, where $x_{i}$, $y_{i}$, $z_{i}$ are the Cartesian coordinates of the *i*-th AA, and $x_{j}$, $y_{j}$, $z_{j}$ are the Cartesian coordinates of the *j*-th AA, then there is a contact between the two AAs at positions *i* and *j*, $\forall i,j\in \bar{1,n}$.

For finding a collision (two AAs in the same place), we use the following equations, where the terms have the same understanding as above:

For 2D square lattice, if i≠j: $\left(x_{i}-x_{j}\right)+\left(y_{i}-y_{j}\right)=0$;

For 3D cubic lattice, if i≠j: $\left(x_{i}-x_{j}\right)+\left(y_{i}-y_{j}\right)+\left(z_{i}-z_{j}\right)=0$

If these equations are satisfied, it indicates that there is a collision between the AAs at positions *i* and *j* in the conformation.

**Figure 5:**
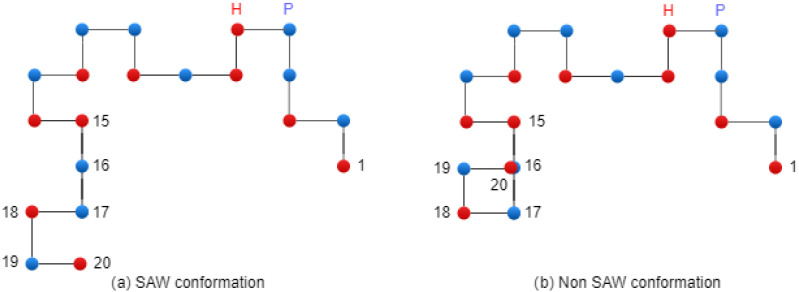
Conformations on 2D Lattice.

Figure 5 presents two conformations: on the left side, there is a SAW conformation, and on the right side, there is a conformation that has one collision (non-SAW conformation).

**Fitness Evaluation Strategy.** The fitness function evaluates how close a given chromosome is to the optimum solution. It determines how fit a chromosome is. We have used a fitness function (see Equation (5)) inspired by the code of Alican Toprak. (https://github.com/alican/GeneticAlgorithm accessed on 6 November 2025).

(5) $$Fitness\left(c\right)=\frac{E(c)\cdot 100+1}{collisions{\left(c\right)}^{2}}$$

where *c* is the conformation, E(c) is the number of contacts, and the collisions(c) is the number of collisions of the conformation. This is computed by checking the topological neighborhood of all AAs on the lattice, according to the number of contacts (Equation (2)) and the number of collisions (collisions(c)). There are two exceptions: if collisions(c)=0 then the formula becomes Fitness(c)=E(c)∗100+1 and if collisions(c)=1 then collisions(c) is replaced with 2. Thus, the fitness increases with the number of contacts and is strongly penalized by the number of collisions.

**Adapted tournament selection.** We proposed an adapted variant of tournament selection, which increases the probability of individuals with low energy values entering the next generation, while implicitly conserving the best individual. Specifically, the selection is applied to the previous population by choosing pairs of chromosomes at random, and after comparing them, the best one is copied into the position of the worst one. This way, the best chromosome is preserved through the generations.

**Figure 6:**
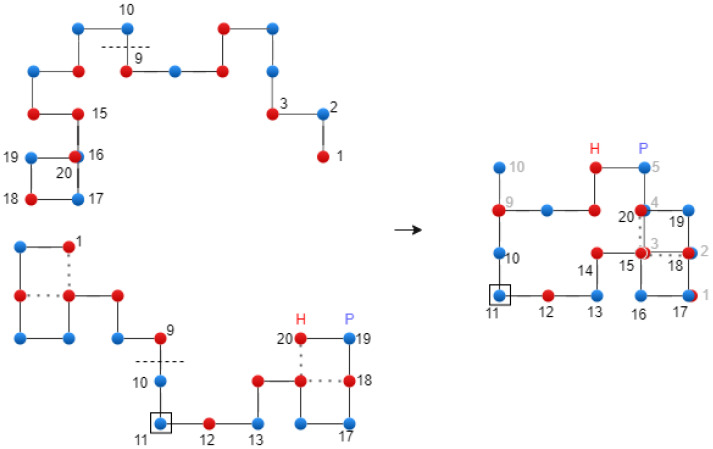
Translational crossover on the 2D Lattice—exemplification.

**Crossover.** In addition to the rotational crossover used in our previous work [49], we apply the translational crossover. For both types of crossover operators, the best chromosome (C∗) from the current population is protected. The crossover operation is performed using the following formula:

(6) $$C\left(t+1\right)=Crossover\left(C\left(t\right),D\left(t\right),C*\right)$$

where C∗ is the best chromosome from the current population, and C(t) and D(t) are parent chromosomes. The reason for introducing C∗, as parameter into the crossover operator is to protect this chromosome. Figure 6 shows the translational crossover.

**Mutation.** We employ translational, rotational, and diagonal mutations. Given a chromosome $C=[d_{1},d_{2},\ldots ,d_{n}]$, where $d_{i}\in \left\{R,U,L,D\right\}$ for 2D (or $d_{i}\in \left\{R,U,L,D,F,B\right\}$ for 3D), it is mutated to a new chromosome, **C’**. To achieve this, a position *g* (1 ≤ g ≤ n), known as the mutation point, is randomly chosen for each conformation. The letter at position *g* is then replaced by one letter sampled uniformly from the set of possible directions.

**Figure 7:**
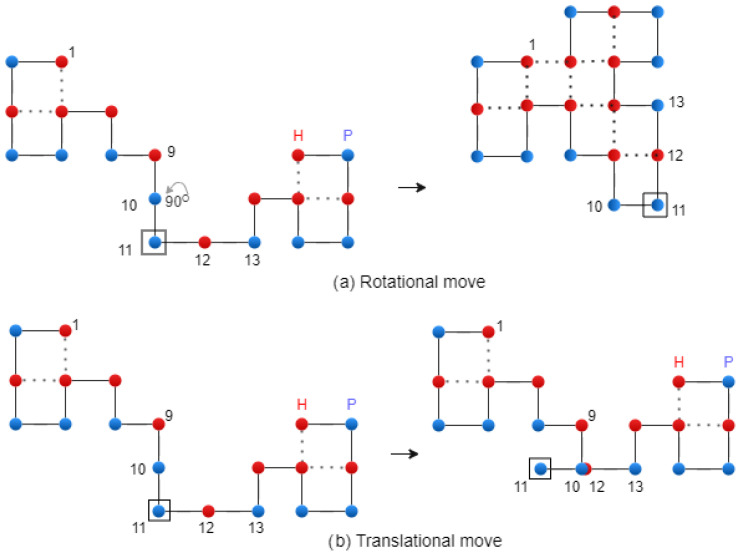
Rotational and translational mutation on the 2D Lattice – exemplification.

For the rotational mutation, the modification is applied to the SRL string, and the next letter after the *g* point remains unchanged. Then, the SRL string is converted to the RULD string. This modification produces a rotation of the second part of the chain by $90^{\circ }$, $180^{\circ }$, or $270^{\circ }$, respectively. In the case of translational mutation, the modification is applied to the RULD string, and the next letter after the *g* point remains unchanged. Figure 7 shows the two types of mutation. A diagonal move is executed on the two letters of the RULD string that form a corner. Finally, the mutation operation is performed using the following formula:

(7) $$C\left(t+1\right)=Mutation\left(C\left(t\right),C*\right)$$

where C∗ is the best chromosome from the current population and *t* represents the iteration number (time). The reason for introducing C∗, as parameter into the mutation operator is to protect it.

**The algorithm.** For every generation, the next operations are executed: (a) rotational crossover, (b) translational crossover, (c) translational mutation, (d) rotational mutation, (e) diagonal mutation and (f) tournament selection. After iterating all generations, the algorithm returns the best conformation obtained.

Pseudocode of the ACGA algorithm skeleton is given in Algorithm 3. As can be seen, the stopping criterion of the algorithm consists of reaching the number of generations, given as an input parameter.
**Algorithm 3** All Conformations Genetic Algorithm (ACGA)1:Input: population_size,generations,HPseq,lattice_type2:Output: C*(RULDstring)3:Initialization of the population $P_{i}\left(i=1,2\ldots n\right)$4:Compute fitness of each conformation conf Equation (5)5:Adapted tournament selection6:C*← the best conformation7:t←08:**while** (t<generations)**do**9:    **for** (every chosen conformation) **do**10:        $C_{t+1}⟵Crossover\left(C_{t},D_{t},C*\right)$11:        $C_{t+1}⟵Mutation\left(C_{t},C*\right)$12:    **end for**13:    Compute the fitness of modified chromosome14:    Adapted_tournament_selection15:    C*⟵ the best conformation16:    t⟵t+117:**end while**18:return C*

### 4.4. Computational Complexity Analysis of ACGA

The computational cost of the ACGA algorithm can be expressed as a function of:

- *n*: number of amino acids in the sequence (length of HP string);
- *P*: population size;
- *G*: number of generations;
- $O_{\mathrm{fit}}$: cost of computing the fitness for a single conformation.

**Time complexity.** In each generation, the algorithm performs the following dominant steps:

1. Fitness evaluation: For each of the *P* individuals, the number of hydrophobic–hydrophobic (H–H) contacts and collisions is computed. In the naive implementation, this requires pairwise checks between amino acids, leading to $O(n^{2})$ time per individual. Thus, the fitness evaluation per generation costs $O(P\cdot n^{2})$.
2. Genetic operators: Crossover and mutation operate on conformation strings of length *n*, requiring O(n) per operation. As a constant fraction of the population is modified in each generation, the total cost for genetic operators is O(P·n), which is asymptotically dominated by the $O(P\cdot n^{2})$ fitness term for large *n*.
3. Selection: The adapted tournament selection compares pairs of individuals, with O(P) comparisons per generation.

Combining these, the overall time complexity is:

$$T_{\mathrm{ACGA}}=O\left(G\cdot P\cdot n^{2}\right)$$

**Space complexity.** The primary memory requirements come from:

1. Storage of the population: *P* individuals, each with a conformation string (O(n)) and auxiliary encodings (absolute, relative) plus Cartesian coordinates (O(n)). This is O(P·n).
2. Temporary arrays for crossover/mutation operations: O(n).

Thus, the total space complexity is:

$$S_{\mathrm{ACGA}}=O\left(P\cdot n\right)$$

In conclusion, for typical parameter settings in protein structure prediction benchmarks (n≤1000, *P* up to $10^{5}$, *G* in the tens of thousands), ACGA remains computationally tractable.

### 4.5. Application Architecture Overview

**Figure 8:**
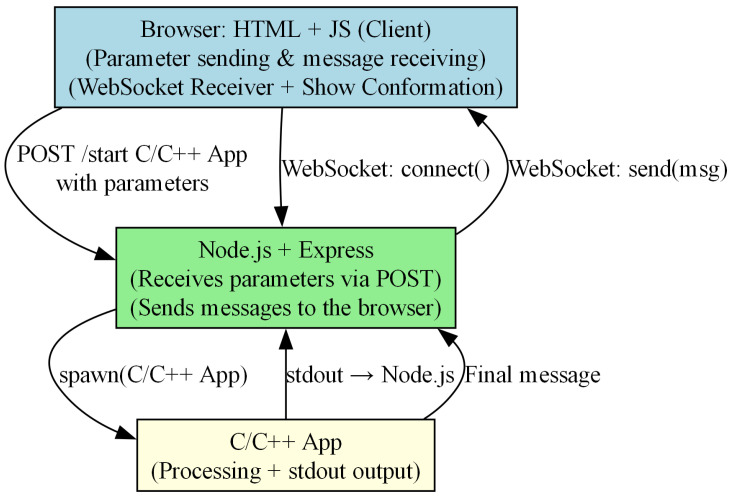
Application architecture involving browser, Node.js server, and C/C++ processing module.

Figure 8 illustrates the high-level architecture of the application, which integrates a web-based front-end (HTML + JavaScript), a middleware server implemented in Node.js, and a back-end component developed in C/C++. The system employs both HTTP and WebSocket communication mechanisms for real-time data exchange.

- The client-side browser component (node A) allows users to send parameters and receive processed messages. It initiates a WebSocket connection with the Node.js server and receives messages that incorporate the best protein conformation for every iteration. Then, a Viewer p5-based shows the conformation (p5.js is a free and open-source JavaScript library [50]).
- The Node.js server (node B) receives input parameters from the browser via an HTTP POST request. It is responsible for spawning the C/C++ application as a separate process and handling message forwarding through WebSocket.
- The C/C++ application (node C) performs the core processing logic (MC, SA, ACGA). Its output is redirected to the Node.js server through standard output (stdout). When the execution is finished, a completion message is sent (and a final message is sent upon completion.)

The communication flow is as follows: the browser sends a POST request to start the processing application with the desired parameters. Node.js spawns the C/C++ executable and relays its output back to the browser using WebSocket messages. A special final message indicates the end of processing.

## 5. Results Analysis

The algorithm has been implemented in the C++ programming language, and tests were conducted on a PC with a Intel(R) Core i7, 2.8 GHz, processor with 4 physical CPU and 4 logical CPU, and 8 GB of RAM running Windows 10. Two implementation variants were developed: one using an OOP style for better software qualities (readability, maintainability, usability) but with slightly poorer execution time performance, and another using the standard C style, which is approximately 40 times faster, mainly due to a more optimized implementation of the selection operation. The results presented in this report are based on the second implementation variant.

To evaluate the efficiency of the algorithm, experiments were conducted on nine popular benchmark sequences with varying lengths ranging from 20 AAs to 85 AAs (see Table 1). The first eight sequences were taken from [9], and the ninth sequence was taken from [16]. The table contains information about the sequence ID, the protein length, and the HP string.

The population size and the number of generations were chosen to allow a wide range of possible conformations to be evaluated. For the selection, mutation, and crossover operators, we tested multiple configurations and adopted the ones that produced the best results after parameter tuning. For the algorithm parameters, we considered various combinations of the following parameters to explore the solution space:

(1) Population size: 10, 50, 100, 300, 500, 1000, 2000, 5000, 10,000, 20,000, 50,000;
(2) Number of generations: 50, 100, 200, 300, 500, 1000, 5000, 10,000;
(3) Translational mutation percent: 0.3;
(4) Rotational crossover percent: 0.4;
(5) Adapted tournament selection percent: 0.35.

For each combination, we repeated the execution 20 times and computed the average energy obtained. We have established 20 considering the aspect to have enough data to have enough data to be able to make simple (like data normality assumption verification) or even advanced statistical analyses (combined analyses where different assumptions must be met).

Table 2 shows the best results obtained for the nine benchmark sequences, which are highly influenced by the population size. The empty value, marked with a “–” in the table, indicates that no data are available in the literature.

**Figure 9:**
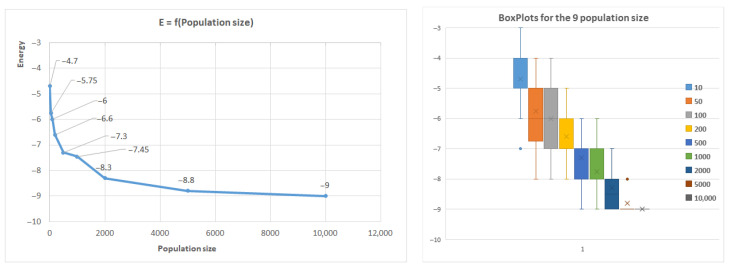
Average energy related to population size in the case of the first benchmark.

Figure 9 further illustrates how the population size affects the results, with the first benchmark sequence of length 20 used as an example. Similar trends were observed for other sequences, where shorter sequences (less than 50 AAs) reached optimal conformations rapidly by increasing the population size, while longer sequences did not reach the optimum.

The algorithm indicates good convergence, as the optimum conformation is typically achieved within 100 generations for small conformations. However, for larger conformations, significant improvements were not observed after 100 generations. The algorithm’s robustness was confirmed by repeating executions with the same parameters 20 times, resulting in a relatively low standard deviation. The box plot in Figure 9 presents the quartile median calculus.

Table 3 and Table 4 show the best conformations obtained by MC, SA and ACGA for the 2D square and 3D cubic lattices, respectively. In conclusion, even for long proteins, the ACGA approach offers a reasonable possibility of finding solutions very close to the optimal ones.

**Algorithms Comparison.** The comparative analysis of the implemented algorithms gave the following observation:

- MC achieved the low energy results (can be seen in the Table 2) and has dynamic search behaviour with exploratory capability. However, due to its fixed temperature, it is prone to high fluctuations and occasional instability.
- SA displayed a similar convergence and stability with MC, and was more resilient to local optima thanks to its cooling schedule. Overall, its results (energy) are slightly better those of MC algorithm.
- ACGA was effective at exploring diverse conformations and maintaining population diversity. Its performance depends strongly on the balance between exploration and exploitation parameters, such as mutation rate and selection pressure. Among the three algorithms, ACGA obtained the best energy for every sequence, as shown in Table 2.

## 6. Discussion

MC proved the advantage of simplicity in implementation and high efficiency in small search spaces. However, the limitations include a large fluctuation that may lead to inconsistent solutions.

SA proved to have increased stability (without extreme oscillations) and achieved better energy compared to MC. Main disadvantage consists of in the fact that MC and SA find conformations with the high energy, far from the energy of optimal conformation.

The ACGA algorithm was efficiently optimized from a computational point of view, enabling the use of large populations to maintain the diversity of individuals in the context of limited computing resources. Experiments have been conducted for several benchmark HP sequences, and the comparative analysis showed that the proposed genetic algorithms offer valuable advantages for PSP on the HP model, providing optimal solutions in most cases, i.e., from a maximum of 20 trials, we give the standard deviation.

In comparison to all these previous results, our approach addresses both 2D square and 3D cubic cases, and we achieved optimum conformations for sequences of length less than or equal to 50. Concerning the 2D square and 3D cubic models, our results are similar for larger sequences.

Unlike HP Protein Visualizer, which prioritises exploratory use and algorithmic approaches through simplified lattice models, AlphaFold and RoseTTAFold aim for highly accurate real-world predictions. While they require substantial computational resources, HP-based tools can be executed in a web browser and are more accessible for didactic and exploratory use.

## 7. Conclusions

The HP model is an ab initio paradigm to model and understand protein folding and is one of the most extensively studied physical models for protein structure prediction from sequences. While the HP model appears very simple, solving it is proven NP-hard. Based on this fact, it is a very good benchmark problem for GA.

We have introduced a novel hybrid Genetic Algorithm that also offers interpretability for PSP that goes beyond considering only the SAW conformations. Instead, our approach allows any conformations in the populations at all stages. This increased flexibility improves the likelihood of obtaining good conformations, even starting from non-SAW ones, as the energy associated with these conformations shows chaotic behavior.

Our primary focus has been on computational efficiency, as it enables reasonable computation times even for large populations. Working with large populations is crucial to achieving the necessary diversity for convergence. The results obtained on a popular benchmark dataset are highly promising, with the optimal solution being achieved in most cases, and for others, the distance from the optimal solution is minimal.

To further improve the algorithm, we plan to explore parallel computation, which can improve computational performance [51], allowing the use of even larger populations. Larger populations further increase the chances of reaching optimal solutions for all cases. Parallelization will be a key focus of our future work. Additionally, we intend to apply the parallel AGCA to real proteins and compare its results with the native conformations from the Protein Data Bank (PDB) [27] and those obtained using the AlphaFold algorithm. This comparison will provide valuable insights into the performance of our algorithm on real biological proteins.

## Figures and Tables

**Table 1:** Benchmark data set.

ID Seq	No of AA	Sequence
1	20	HPHP PHHP HPPH PHHP PHPH
2	24	HHPP HPPH PPHP PHPP HPPH PPHH
3	25	PPHP PHHP PPPH HPPP PHHP PPPH H
4	36	PPPH HPPH HPPP PPHH HHHH HPPH HPPP PHHP PHPP
5	48	PPHP PHHP PHHP PPPP HHHH HHHH HHPP PPPP HHPP HHPP HPPH HHHH
6	50	HHPH PHPH PHHH HPHP PPHP PPHP PPPH PPPH PPPH PHHH HPHP HPHP HH
7	60	PPHH HPHH HHHH HHPP PHHH HHHH HHHP HPPP HHHH HHHH HHHH PPPP HHHH HHPH HPHP
8	64	HHHH HHHH HHHH PHPH PPHH PPHH PPHP PHHP PHHP PHPP HHPP HHPP HPHP HHHH HHHH HHHH
9	85	HHHH PPPP HHHH HHHH HHHH PPPP PPHH HHHH HHHH HHPP PHHH HHHH HHHH HPPP HHHH
		HHHH HHHH PPPH PPHH PPHH PPHPH

**Table 2:** Best energy for 2D and 3D square lattice for the 9 considered benchmarks.

ID Seq	Length	Optimal 2D	ACGA 2D	MC 2D	SA 2D	Optimal 3D	ACGA 3D	MC 3D	SA 3D
1	20	−9	**−9**	**−9**	**−9**	−11	**−11**	**−11**	**−11**
2	24	−9	**−9**	**−8**	**−8**	−13	**−13**	−10	−11
3	25	−8	**−8**	−7	−6	−9	**−9**	−8	**−9**
4	36	−14	**−14**	−10	−10	−18	**−18**	−15	−15
5	48	−23	−22	−18	−18	−31	**−31**	−27	−28
6	50	−21	**−21**	−16	−17	−34	−31	−30	−30
7	60	−36	−35	−28	−30	−55	−49	−42	−42
8	64	−42	−38	−32	−32	−59	−49	−45	−46
9	85	−53	−48	−42	−43	−	−73	−62	−67

**Table 3:** 2D conformations obtained by ACGA for the benchmark sequences.

ID Seq	Optimal Conformations on 2D Square HP Model
1	ULLDRDLDLDRRURDRUUL
2	LULDLDRDRURRRDRURULULDL
3	RULURUUURDDRURDDLDRDLLUU
4	LDLDRDLDDLLURULLURRUULDLUULDDDLDRDD
5	RURDRURDRRRDLDLULDLULDLULDLDRRRDLDRRRRURULLLDR
6	RDLLUURURDRURDRDLLDDLLDLLLLLLUULURRURDDLDRRURUULD
7	URDDDRUURDDDLDRRUUUUULLURRURRRRDLDLULDDRRRURDDLLLLDRRDLLDRR
8	RDLLULDLULDLUURURDRURDRRRDRDLDRDLDRDLDLULDLULDDLUUURRRRULLLURRR
9	RRRRULLLURULLDDLULDLLDLUUURDRURRURRUUULDDLLDLULDLLDLUURRURRRRULLLLLLURURDRURDRURRDL

**Table 4:** 3D conformations obtained by ACGA for the benchmark sequences.

ID Seq	Optimal Conformations on 3D Square HP Model
1	DLLURUFDLDRRUUURDBL
2	RRULUBDRDLDLUBUFULDLFRR
3	RBRULUURBLDDRDLLUULLFRRD
4	FLLLDRFLLLURURDRFDLULURRURDBLBULDLL
5	DRDRURRULURULLLDDRUFRDLDRFFLLURBRRULLULDLBRDFDB
6	RDLLULFDRURDRURULLUBDLLUFRDLFDRUURDRDLDLFRRUULLDR
7	RDLFRDLDRRURDDBLURULLLDRDLLUUUBRRDLDRRRULBDRDLLUUULDDDFRDLD
8	BRRULLFRDRURDDLLFURRULLULDLDRDDFURDRURULLLURRRULLFFDRDLDRBUULDD
9	FFLDLBRRDRRUULDBURDDDRFLFULLURURDRUBDRFFLULDLDLLURULURRDFRDLDLUULDDLFRULURRURDDRDLLU

## Data Availability

The data set used in the study was taken from Unger1993 [9] and Huang2010 [16]. The conformations achieved by the ACGA on the 2D square lattice and the 3D cubic lattice can be visualized in the files “Experiments.xlsx” and “Experiments3D.xlsx”.
